# Health-related quality of life in severe hypersensitivity reactions: focus on severe allergic asthma and hymenoptera venom anaphylaxis—a cross-sectional study

**DOI:** 10.3389/fpsyg.2024.1394954

**Published:** 2024-08-23

**Authors:** Luisa Ricciardi, Orlando Silvestro, Gabriella Martino, Antonino Catalano, Carmelo Mario Vicario, Trine Lund-Jacobsen, Peter Schwarz, Daniela Sapienza, Sebastiano Gangemi, Giovanni Pioggia, Concetto Mario Giorgianni

**Affiliations:** ^1^Department of Clinical and Experimental Medicine, University of Messina, Messina, Italy; ^2^Department of Health Sciences, University Magna Graecia of Catanzaro, Catanzaro, Italy; ^3^Department of Cognitive Science, Psychology, Education and Cultural Studies, University of Messina, Messina, Italy; ^4^Department of Endocrinology and Metabolism, Rigshospitalet, Copenhagen, Denmark; ^5^Department of Biomedical and Dental Sciences and Morphofunctional Imaging, University of Messina, Messina, Italy; ^6^Institute for Biomedical Research and Innovation (IRIB), National Research Council of Italy (CNR), Messina, Italy

**Keywords:** clinical psychology, immunology, alexithymia, severe allergic asthma, hymenoptera venom anaphylaxis, H-R quality of life, severe hypersensitivity reactions, outdoor workers

## Abstract

**Background:**

Growing evidence reveals the important role of clinical psychological factors in chronic-immune diseases. The aim of this study was to investigate Health-Related Quality of Life (HR-QoL), depression, anxiety, and alexithymia in patients with severe hypersensitivity reactions such as Severe Allergic Asthma (SAA) and Hymenoptera Venom Anaphylaxis (HVA).

**Methods:**

The Short-Form Health Survey-36 (SF-36), the Beck Depression Inventory Questionnaire (BDI-II), the Hamilton Anxiety Rating Scale (HAM-A) and the Toronto Alexithymia Scale (TAS-20) were used to assess HR-QoL and clinical psychological features of patients with SAA and HVA.

**Results:**

Overall, 78 patients were recruited. Patients with SAA (*n* = 35) reported lower scores for physical functioning [65 (58–75) vs. 90 (85–95); *p* = <0.001], role limitations due to physical health [25 (0–50) vs. 62 (50–75); *p* = 0.004], bodily pain [47.5 (41.1–61.3) vs. 55.5 (55–96); *p* = 0.001], general health [40 (30–60) vs. 70 (50–80); *p* = 0.0003] and social functioning [50 (37.5–62.5) vs. 62.5 (54.9–75); *p* = 0.007] while higher scores for depressive symptoms [14 (11–15.4) vs. (9.5 (6–15.4); *p* = 0.05)] compared to HVA patients (*n* = 43). All the dimensions of SF-36 were negatively correlated with anxiety (*r* from −0.26 to −0.66; *p*^all^ < 0.01) and depressive symptoms (*r* from −0.44 to −0.73; *p*^all^ < 0.001). Alexithymia was negatively correlated with vitality (*r* = −0.28; *p* = 0.02) and mental health (*r* = −027; *p* = 0.03). Additionally, patients with alexithymia (38% of participants) showed higher levels of depressive symptoms [9.5 (10–19) vs. 14 (6–13.9); *p* = 0.005] and anxiety levels [31 (27.9–35) vs. 24 (16–33.9); *p* = 0.02]; they also showed less vitality [40 (39.9–50) vs. 55 (50–60) *p* = 0.01], social functioning [50 (37.5–62.5) vs. 62.5 (50 vs. 75); *p* = 0.01] and mental health [48 (44–60) vs. 68 (56–76); *p* = 0.004].

**Conclusion:**

Clinical psychological features due to severe hypersensitive reactions may contribute to the patient’s perceived HR-QoL. Focused clinical psychological interventions should be promoted to improve the clinical management of such conditions.

## 1 Introduction

Empirical evidence suggests that chronic diseases may impact psychological well-being, increasing the risk of morbidity with incident psychopathologies, such as depression and anxiety (Di Giuseppe and Conversano, 2022; Isvoranu et al., 2021; Lin et al., 2022). Chronic illness represents a challenge for patients and requires continuous adjustment in the management of daily life (Merlo, 2019; Braido, 2013; van Houtum et al., 2015).

Asthma is a chronic respiratory disease characterized by persistent airway inflammation and airway stiffening, resulting in variable difficulty in breathing (World Health Organization, 2019). Asthma appears with heterogeneous and variable symptomatology, including airway secretion, shortness of breath, wheezing, chest pain, and coughing attacks (Braido, 2013; Louis et al., 2023). For these reasons the Global Initiative for Asthma (2023) classifies asthma based on the control of symptoms and distinguishes between well-controlled or uncontrolled asthma, the latter is characterized by the presence of frequent symptoms’ exacerbations, requiring timely use of oral corticosteroids (OCS) or hospitalization.

Severe Asthma (SA) is a subtype of difficult-to-treat asthma, characterized by poor symptoms control despite adherence to a correctly prescribed maximal inhaler with high-dose inhalant corticosteroids (ICS) and beta2 long-acting agents’ (LABA) treatment (Bagnasco et al., 2021; Ricciardi et al., 2023). Patients with SA represent about 5–10% of the asthma population (Chung et al., 2014). SA is characterized by low lung function, immune dysregulation, and frequent exacerbations, with life-threatening asthma attacks (Backman et al., 2019). For these reasons, asthma is one of the major public health issues worldwide and causes a significant social and economic burden (Mortimer et al., 2022; López-Tiro et al., 2022). Severe allergic asthma (SAA) patients have chronic allergic asthma due to immunoglobulin E (IgE)-mediated sensitization to inhalant allergens, prevalently Dust Mites or, in Southern Italy, to *Parietaria Judaica* pollens (Liotta et al., 2023).

In this context we would mention the *not-negligible* incidence of asthma in workers, as occupational asthma represents an important environmental workplace disease, especially in outdoor workers. Several organic, inorganic and xenobiotic substances may provoke immunoreactions with consequences on the respiratory system, even resulting in occupational asthma (Toletone et al., 2016; Ruëff et al., 2023). Previous literature has also highlighted how occupational asthma influences workers’ perceived quality of life of also concerning syndromes characterized by depression and anxiety (Suarthana et al., 2023; Del Roio et al., 2023).

Gruffydd-Jones et al. (2019) in a multinational survey, analyzing 1.598 patients with poor control of asthma symptoms despite long-term treatment, highlighted a significant reduction in work productivity with a negative impact on emotional well-being at work. Empirical evidence suggests that SA and occupational asthma are associated with low perceived Health-Related Quality of Life (HR-QoL) (Hossny et al., 2017; Bagnasco et al., 2021; Wang et al., 2020), psychological distress (Patella et al., 2022) and significant risk of psychiatric comorbidity, such as anxiety and depression (Roche et al., 2022). Additionally, recent research (Silvestro et al., 2023; Ricciardi et al., 2023) suggests that patients with SA and SAA may present a low awareness to identify and describe one’s feelings, experiencing a clinical condition known as alexithymia which negatively impacts the disease course (Nemiah et al., 1976).

Hymenoptera venom allergy is a typical IgE-mediated disease, provoked by bees or wasps, causing several symptoms including urticaria, angioedema, flushing, itching, hypotension, and anaphylaxis (Sturm et al., 2018): a systemic allergic reaction due to HVA may develop within minutes from a sting and in some cases, it can be potentially life-threatening leading to cardiorespiratory arrest (Schiener et al., 2017). Although there is a lack of epidemiological data, a European multicenter study estimated that about 48.2% of severe hypersensitivity reactions in adults (>18 years old) are caused by insect stings (Worm et al., 2014). Additionally, Pastorello et al. (2001) reported a death range per year due to HVA from 0.03 to 0.48% out of a population of 1,000,000 subjects.

Patients who have experienced anaphylaxis following Hymenoptera stinging have been found to present psychological distress (Schoeben et al., 2020; Ricciardi et al., 2018; Giorgianni et al., 2024). HVA may cause, fear of being stung again by an insect, anxiety, uncertainty, restrictions in social life and everyday activities, therefore leading to a lower HR-QoL (Höfer et al., 2023; Schaarschmidt et al., 2018).

Few study have investigated HVA related to the role of occupational exposure, but it would be appropriate to provide useful information to high risk outdoor workers to correctly manage environmental exposure to hymenoptera stings. Observational studies on populations of HVA subjects have documented that outdoor workers have greatest risk of occasional hymenoptera (Toletone et al., 2016). Since little evidence exists about the crucial role of clinical psychological factors in patients with severe hypersensitivity reactions, the present research study aims to: (1) investigate HR-QoL and clinical psychological characteristics in patients with a history of severe allergic diseases, including SAA and HVA; (2) analyze the clinical-psychological differences between SAA and HVA and (3) evaluate the associations of clinical-psychological features in these severe allergic diseases.

A greater understanding of the psychological complexity of patients suffering from SAA and HVA could promote a greater awareness of the integrated approach needed in such chronic diseases.

## 2 Methods

### 2.1 Participants

This cross-sectional study included 78 patients with a history of severe hypersensitivity reactions, 35 with SAA and 43 with HVA, who referred to the Outpatients Clinic for Allergy and Clinical Immunology at the Department of Clinical and Experimental Medicine of the University Hospital of Messina, Italy between January and May 2022. All SAA patients were treated with Omalizumab, an anti-IgE biologic treatment, in addition to high dose ICS + LABA, while HVA patients were under venom allergen immunotherapy (VAIT), 6 with bee venom extract and 37 with wasp venom extract (ALK, Alutard, Milan). Exclusion criteria were age under 18 years, cognitive decline, moderate to severe kidney or liver failure, heart failure with NYHA (New York Heart Association) class ≥2, cancer, malabsorption, endocrine disorders of thyroid, parathyroid or adrenal glands, and known psychopathological diseases and psychotropic drugs assumption.

### 2.2 Ethics statement

The research will be carried out following the Declaration of Helsinki (World Medical Association, 2013). Ethical approval was obtained from the Ethics Committee of the University Hospital of Messina University (Protocol number 16/19). All patients were informed about privacy and the use of anonymous data for research purposes and signed an informed consent according to the European General Data Protection Regulation 2016/679.

### 2.3 Clinical evaluation

A psychological assessment was conducted by a researcher in clinical psychology, performing a gold-standard clinical psychological interview and a psycho-diagnostics examination.

The Beck Depression Inventory, second edition (BDI-II), was administered to measure depressive symptoms (Beck et al., 1996; Ghisi et al., 2006). It consists of a self-report instrument, composed of 21 items scored on a four-point Likert scale. BDI-II detects both somatic-affective (e.g., agitation, sleep disorder, and loss of energy) and cognitive (e.g., pessimism, suicidal thoughts, self-criticalness) features of depression. In the present study, the Italian version of BDI-II was adopted, as it showed good proprieties and a bi-factorial structure with an *α* coefficient of 0.86 for cognitive/affective and 0.65 for somatic symptoms (Montano and Flebus, 2006).

The Hamilton Anxiety Rating Scale (HAM-A) was used to assess the severity of anxiety symptoms (Hamilton, 1959). It is a self-report questionnaire, consisting of 14 items scored on a five-point Likert scale. HAM-A presents a bi-factorial structure, detecting both somatic (e.g., respiratory, autonomic, gastrointestinal symptoms) and psychic components of anxiety (e.g., tension, fears and depressed mood). Previous research documented, good statistical proprieties, high reliability, and diagnostic accuracy for the Italian version of this scale (Martino et al., 2021; Scimeca et al., 2014).

The Italian version of the Short-Form Health Survey-36 (SF-36) was used to measure patients’ perceived HR-QoL. SF-36 is a self-administered questionnaire that detects eight dimensions of HR-QoL, comprising physical functioning, limitation due to physical role, bodily pain, general health, vitality, mental health, limitation due to emotional role and social functioning (Ware and Sherbourne, 1992). SF-36 presents a range score from 0 to 100; a lower score indicates a poorer HR-QoL. Previous studies confirmed the psychometric qualities of the scale on Italian clinical samples, with reliability (Cronbach’s *α*) for the eight factors ranging from 0.77 to 0.93 (Apolone and Mosconi, 1998).

The Toronto Alexithymia Scale-20 (TAS-20) was used to assess alexithymic traits (Taylor et al., 1997; Taylor, 2000; Bagby et al., 1994a). It consists of a self-report questionnaire, composed of 20 items scored on a five-point Likert scale. Three cut-off scores were identified to recognize alexithymic (≥61), borderline (51 to 60) and non-alexithymic (≤50) individuals (Bagby et al., 1994b). TAS-20 presents a three-factor structure that comprises the main features of alexithymia: difficulty identifying feelings (DIF), difficulty describing feelings (DDF) and externally oriented thinking (EOT). In the present study, the Italian version of TAS-20 was used; previous research highlighted a good propriety and reliability of the three factors structure (Cronbach’s *α*) considering a score of 0.86 for full scale and 0.83, 0.79, and 0.81 for DIF, DDF, EOT, respectively (Bressi et al., 1996).

### 2.4 Statistical analyses

Statistical analysis was performed using MedCalc software (version 10.2.0.0; Mariakerke, 173 Belgium). The Kolmogorov–Smirnov test was used to verify the normal distribution of values of studied variables. The entire sample was divided into groups according to the allergic disease and the presence (TAS-20 scores ≥61) or the absence of alexithymia and the Mann–Whitney test was used to observe any differences in the level of anxiety, depressive symptoms and QoL. The *χ*^2^ test was used to calculate differences in the proportion of categorical variables. Spearman correlation coefficient was applied to evaluate the degree of association between two variables. Finally, a multiple regression analysis was performed to explore the association between alexithymia, identified as the dependent variable, and the explanatory variables age, age at diagnosis, anxiety and depression. For all the tests used, the value of *p* < 0.05 was considered to indicate the statistical significance.

### 2.5 Results

**Figure 1:**
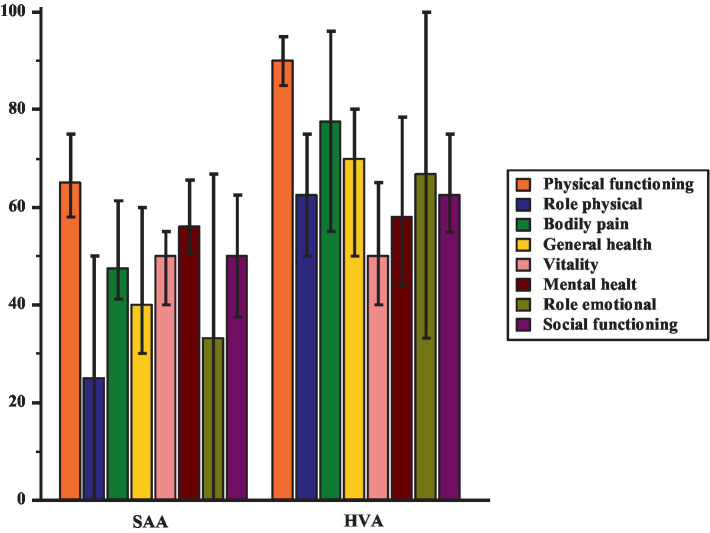
Difference in HR-QoL between group with SAA and group with HVA.

The main socio-demographic and clinical characteristics of recruited patients are reported in Table 1. Overall, the median age of participants was 55 yrs., and the prevalent gender was male (57%). Participants showed moderate anxiety levels, mild depressive symptoms, conceivable alexithymia and low perceived HR-QoL. The age at diagnosis was significantly higher in patients with HVA in comparison with patients with SAA [44 (38–48.2) vs. 21 (13–35), *p* = 0.002, respectively]. Moreover, the HVA group presented a significant difference in the employment status in comparison with patients with SAA, particularly in the number of full-time workers (84% vs. 54%) respectively. Regarding clinical psychological features, both groups showed no significant differences in anxiety and alexithymia levels. However, the SAA group presented higher depressive symptoms levels in comparison with HVA patients [14 (11–15.4) vs. (9.5 (6–15.4); *p* = 0.05)]. On the other hand, patients in the HVA group showed higher levels of externally oriented thinking [25 (23–28) vs. 23 (20–25); *p* = 0.05]. Data on the SF-36 survey are reported in Figure 1; patients with SAA showed a lower score in physical functioning [65 (58–75) vs. 90 (85–95); *p* < 0.001], role limitations due to physical health [25 (0–50) vs. 62 (50–75); *p* = 0.004], bodily pain [47.5 (41.1–61.3) vs. 55.5 (55–96); *p* = 0.001], general health, [40 (30–60) vs. 70 (50–80); *p* = 0.0003] and social functioning [50 (37.5–62.5) vs. 62.5 (54.9–75); *p* = 0.007] in comparison to the HVA group. There were no statistically significant differences between the groups concerning vitality, role limitations due to emotional problems and mental health. Considering TAS-20 scores ≥61, it was observed that 29 (38%) participants exhibited clinically significant levels of alexithymia. Taking into account the presence (TAS-20 scores ≥61) or absence (TAS-20 <61) of alexithymia, it was highlighted that patients with alexithymia presented higher levels of depression [9.5 (10–19) vs. 14 (6–13.9); *p* = 0.005], anxiety, with particular regard to the psychic dimension [31 (27.9–35) vs. 24 (16–33.9); *p* = 0.02] and lower scores in vitality [40 (39.9–50) vs. 55 (50–60); *p* = 0.01], social functioning [50 (37.5–62.5) vs. 62.5 (50 vs. 75); *p* = 0.01] and mental health [48 (44–60) vs. 68 (56–76); *p* = 0.004] to the patients without alexithymia.

**Table 1:** Main socio-demographic and clinical characteristics of participants.

	Total (*n* = 78)	Severe allergic asthma (*n* = 35)	Hymenoptera anaphylaxis (*n* = 43)	*p*
Age (yr)	55 (50–59)	59 (54–64)	52 (46–57)	0.05
Age at diagnosis (yr)	38 (25–45)	21 (13–35)	44 (38–48.2)	0.002*
BMI (Kg/m^2^)	26.3 (24.7–27.9)	27 (24–30)	25.8 (24.6–27.3)	0.4
Gender [male (%)]	45 (57)	13 (37)	32 (74)	0.002*
Education				
Primary school [*n* (%)]	1 (1)	1 (2)	0(0)	
Secondary school [*n* (%)]	17 (22)	7 (20)	10 (23)	
High school [*n* (%)]	46 (59)	19 (56)	27 (63)	
Bachelor’s degree [*n* (%)]	13 (17)	7 (20)	6 (14)	
PhD or specialization [*n* (%)]	1 (1)	1(2)	0(0)	0.52
Marital status				
Single [*n* (%)]	17 (22)	8 (22)	9 (21)	
Married [*n* (%)]	56 (72)	24 (69)	32 (74)	
Divorced [*n* (%)]	3 (4)	1 (3)	2 (5)	
Widower [*n* (%)]	2 (2)	2 (6)	0 (0)	0.43
Employment status				
Full time [*n* (%)]	55 (70)	19 (54)	36 (84)	
Unemployed [*n* (%)]	7 (9)	4 (11)	3 (8)	
Pensioner [*n* (%)]	12 (16)	10 (29)	2 (4)	
Housewife [*n* (%)]	4 (5)	2 (6)	2 (4)	0.01*
Anxiety levels				
HAMA score	29 (25–32.6)	31 (23–36)	28 (20.8–33.6)	0.39
HAMA somatic symptom score	14 (11–16)	15 (12–17)	12 (8.4–16.2)	0.13
HAMA psychic symptom score	15 (13.4–16)	15 (12–17.4)	15 (13.4–17)	0.89
Depression severity				
BDI-II score	12 (9.4–14)	14 (11–15.4)	9.5 (6–14.6)	0.05*
Alexithymia				
TAS-20 total score	59 (55–62)	56 (45–63)	61 (56–65)	0.12
DIF	19 (16–22)	18 (13–23)	19.5 (16–22)	0.48
DDF	15 (13.3–16)	15 (11–16)	16 (13–18)	0.29
EOT	24 (23–25.6)	23 (20–25)	25 (23–28)	0.05*

Values are expressed as median (IQR). HAMA-A, Hamilton anxiety rating scale; BDI-II, Beck Depression Inventory II Edition; TAS-20, Toronto Alexithymia Scale 20-item; DIF, difficulty identifying feelings; DDF, difficulty describing feelings; EOT, externally oriented thinking.

Overall, the number of explored clinical psychological morbidities was positively related to age (*r* = 0.48; *p* < 0.01), BMI (*r* = 0.34; *p* = 0.003) anxiety (*r* = 0.32; *p* = 0.01), depression (*r* = 025; *p* = 0.04); conversely, it was negatively associated with HR-QoL: physical functioning (*r* = −036; *p* = 0.004), limitations due to physical health (*r* = −0.37; *p* = 0.003), bodily pain (*r* = −0.35; *p* = 0.005) and general health (*r* = −0.38; *p* = 0.003). At correlation analysis, all the dimensions of SF-36 were negatively associated with age (*r* from −0.2 to −0.57; *p*^all^ < 0.05), anxiety, both somatic and psychic (*r* from −0.26 to −0.66; *p*^all^ < 0.01) and depressive symptoms (*r* from −0.44 to −0.73; *p*^all^ < 0.001). Similar associations were observed when analyzing these variables in both groups separately. Moreover, considering the entire sample, TAS-20 was negatively correlated with vitality (*r* = −0.28; *p* = 0.02) and mental health (*r* = −027; *p* = 0.03), also DDF was negatively correlated with vitality (*r* = −028; *p* = 0.02) and mental health (*r* = −0.32; *p* = 0.01) and positively associated with depressive symptoms (*r* = 0.26; *p* = 0.04). Additionally, anxiety (somatic and psychic) was positively correlated with depressive symptoms (*r* = 0.61; *p* < 0.001). Only in the group of patients with SAA, the alexithymia total score was associated with anxiety (*r* = 0.36; *p* = 0.04), while DDF was correlated with depressive symptoms (*r* = 0.42; *p* = 0.01).

Finally, at multiple regression analysis which considered alexithymia as the dependent variable, and age, age at diagnosis, anxiety and depression as explanatory variables, only anxiety was significantly and independently associated with alexithymia in the SAA group (*β* = 0.4375; SE = 0.198; *p* = 0.03).

## 3 Discussion

This research highlights that patients with a history of severe allergic diseases, including SAA and HVA, show moderate anxiety levels, mild depressive symptoms, conceivable alexithymia and low perceived HR-QoL. The main results of this study indicate that the group of patients with SAA showed low HR-QoL in both physical and emotional dimensions. In line with this statement, empirical evidence underlined that the experience of living with SAA significantly impacts physical and emotional functioning, determining limitations in completing daily activities (McDonald et al., 2018), decrease in work performance (Hiles et al., 2018), and difficulty in managing leisure time and social activities (Dockrell et al., 2007).

Regarding differences between patients with SAA and with HVA, the first group reported lower results in all dimensions of the SF-36, except for vitality, limitations due to emotional role, and mental health. Both groups reported low-to-medium scores in these dimensions, highlighting the potential negative impact of these chronic conditions on patients’ psychological well-being. Concerning these results, we hypothesized that, if on the one hand the invalidating respiratory symptoms of SAA, most of all shortness of breath, may cause significant impairment in all dimensions of perceived HR-QoL, on the other hand, anaphylaxis experienced by patients with HVA can lead to uncertainty and fear of experiencing a new hypersensitivity reaction, with negative effects on psychological dimensions of HR-QoL in the long term. Tal et al. (2020), analyzing 44 patients with a history of anaphylaxis due to a Hymenoptera sting, highlighted a major susceptibility to symptoms of post-traumatic stress disorders in patients with local reactions, demonstrating that severe hypersensitivity reactions could compromise psychological health.

In the present study, no differences were found between the two disease groups for anxiety levels and alexithymia. However, patients with SAA presented more depressive symptoms than patients with HVA. Numerous evidence suggests comorbidity between asthma and depression (Zielinski et al., 2000; Gao et al., 2015; Lu et al., 2018). Meta-analysis of Jiang et al. (2014) highlighted that patients with depression are more likely to present in comorbidity asthma (3.17 times more) than healthy subjects. Similarly, patients with asthma are more likely to have depression as a comorbidity (1.52 times more). According to Medina-Rodriguez et al. (2018), patients with major depressive disorder present immune system alterations with significant changes in plasma cytokine levels. Therefore, the inflammatory response might represent a common pathway between allergic diseases such as asthma and mental disorders (Jiang et al., 2014; Lu et al., 2018).

Regarding the association between clinical psychological features and levels of HR-QoL, depression and anxiety, both somatic and psychic, were related to low HR-QoL, while higher levels of alexithymia were associated with reduced vitality and social function dimensions, in both groups. These findings confirmed the relevance of accurate psycho-diagnostic assessment to detect the presence of clinically significant features in both SAA and HVA patients (Conversano and Di Giuseppe, 2021; Adelmeyer et al., 2021; Esmaeel and Aly, 2019). A multidisciplinary clinical approach, both medical and psychological, could allow early detection of at-risk situations or identification of cases in which a mental disorder is already concluded, to ensure the most suitable patient-tailored treatment (Majellano et al., 2019; Cooley et al., 2022).

Furthermore, it would be appropriate to give indications on the correct risk management at work of subjects highly exposed to hymenoptera stings, both in the case of workers suffering from occupational asthma and for those with HVA. The risk of being affected by the exposure to environmental factors can be the cause of suffering among outdoor workers with the consequent development of depression and anxiety. In the coming researches it could be relevant to study further populations of outdoor workers in relation to what has emerged from our findings to provide guidelines on the management and prevention of such sufferings (Ruëff et al., 2023).

Depression, anxiety, and alexithymia have been associated with adverse health outcomes in several chronic conditions; regarding asthma, in the United States, Lin et al. (2022) highlighted that 14.16% of patients with asthma showed comorbidity with depression and the co-occurrence of asthma and depression was associated with major death risk than in asthmatic patients without depression. The presence of anxiety and/or depression in comorbidity with asthma was associated with poorer asthma control (Stubbs et al., 2022), higher corticosteroid dosage (McLoughlin and McDonald, 2021), greater frequency of exacerbation (Zhang et al., 2016) and greater healthcare use (Sastre et al., 2018). Regarding the correlation of HVA with depression and anxiety, Schaarschmidt et al. (2018), analysing 55 patients with HVA, found that 14.5% showed clinically significant levels of anxiety, while 5.5% had definite depressive disorder. Schoeben et al. (2020) suggested that female patients with HVA had higher anxiety symptoms than male patients. Despite such empirical evidence, there is a lack of knowledge regarding the risk of depression and anxiety in patients with HVA and the impact of these variables on HR-QoL. Therefore, future research should analyse, also through longitudinal designs, the association between psychological factors and HVA, with particular attention to patients who have experienced a severe anaphylactic reaction. The latter, due to the risk of death from anaphylaxis, could develop strong anxiety in outdoor environments, limit social activities and experience low perceived HR-QoL, with negative effects on global psychophysical well-being (Höfer et al., 2023; Nowak et al., 2015).

Regarding alexithymia, in the present study, 38% of participants showed a clinically significant score of TAS-20. Alexithymic patients showed greater depressive symptoms, anxiety, both psychic and somatic, and a lower perception of HR-QoL for the dimension vitality, social functioning, and mental health than non-alexithymic patients. From a qualitative perspective, these findings support the hypothesis that alexithymia is linked to precise medical domains (Tesio et al., 2018; Di Giuseppe and Conversano, 2022), as in the case of dermatological (Holmes et al., 2022; Namdar and Kurtoglu, 2021), gastrointestinal (Fuchs et al., 2023; Martino et al., 2020, 2023; Kano et al., 2018) and respiratory diseases (Silvestro et al., 2023; Ricciardi et al., 2023), as in the present study which considered also patients with a history HVA. Although in the present study, it was not possible to consider causal associations between alexithymia and the other variables, the results allow us to observe how the difficulty in identifying and recognizing feelings and the external event oriented-thinking style may negatively impact the course of chronic illnesses. Alexithymia could alter patients’ ability to distinguish kinaesthetic sensations related to emotional arousal from symptoms of severe hypersensitivity reactions, negatively affecting the ability to self-manage chronic conditions (Baiardini et al., 2011).

Finally, we acknowledge that the present study has some limitations, as the number of participants was low and moreover, that the group with HVA was mostly of men; these factors limit the possibility of generalizing our results to a broader context. The cross-sectional design does not allow us to establish a cause-effect association among clinical psychological features and dimensions of perceived HR-QoL, and because of the lack of studies, the psychological characteristics of patients with HVA should be further investigated. Nevertheless, our findings may contribute to a better understanding of the psychological complexity of patients with hypersensitivity reactions, allowing a greater awareness of how anxiety, depressive symptoms, and alexithymia can influence the clinical characteristics of severe allergic diseases.

## 4 Conclusion

The present study highlights that patients with SAA showed lower perceived HR-QoL and greater depressive symptoms than patients with HVA. Patients with HVA reported moderate-low levels of vitality, limitations due to emotional role, and mental health, as well as patients with SAA. Overall, clinically significant levels of alexithymia were associated with higher depressive symptoms and lower vitality and mental health. Therefore, clinicians and psychologists could apply a multidisciplinary approach, considering the body–mind complex, to early detect clinical psychological features such as depression, anxiety, and alexithymia, and to reach a timely diagnosis and accurate therapeutic support in clinical settings, targeting a higher perceived HR-QoL.

## Data Availability

The raw data supporting the conclusions of this article will be made available by the authors, without undue reservation.
