# Supplementary material for: Health-related quality of life in severe hypersensitivity reactions: focus on severe allergic asthma and hymenoptera venom anaphylaxis—a cross-sectional study
Source: Front Psychol. 2024 Aug 23;15:1394954. doi: 10.3389/fpsyg.2024.1394954 (PMC11377323; doi:10.3389/fpsyg.2024.1394954)
Supplement: Supplementary file 1 [file Data_Sheet_1.PDF]

Supplemetary Table 1 – Multiple regressione analysis

Dependent Y	Alexithymia
Select	SAA

Method	Forward
Enter variable if P<	0,05
Remove variable if P>	0,1

Sample size	33
Coefficient of determination R^2	0,1352
R^2-adjusted	0,1073
Multiple correlation coefficient	0,3677
Residual standard deviation	12,2351

Regression Equation

Independent variables	Coefficient	Std. Error	t	P
(Constant)	42,2185			
HAM-A	0,4375	0,1987	2,202	0,0353

Variables not included in the model
Age
Age at diagnosis
BDI-II

Analysis of Variance

Source	DF	Sum of Squares	Mean Square
Regression	1	725,5324	725,5324
Residual	31	4640,6494	149,6984

F-ratio	4,8466
Significance level	P=0,035
